# Genome-Wide Identification and Characterization of the WRKY Gene Family in *Scutellaria baicalensis* Georgi under Diverse Abiotic Stress

**DOI:** 10.3390/ijms23084225

**Published:** 2022-04-11

**Authors:** Caijuan Zhang, Wentao Wang, Donghao Wang, Suying Hu, Qian Zhang, Zhezhi Wang, Langjun Cui

**Affiliations:** Key Laboratory of the Ministry of Education for Medicinal Resources and Natural Pharmaceutical Chemistry, National Engineering Laboratory for Resource Development of Endangered Crude Drugs in Northwest of China, Shaanxi Normal University, Xi’an 710062, China; cjzhang_@snnu.edu.cn (C.Z.); wangwentao@snnu.edu.cn (W.W.); wangdonghao@snnu.edu.cn (D.W.); husuying@snnu.edu.cn (S.H.); zq182568@snnu.edu.cn (Q.Z.)

**Keywords:** *Scutellaria baicalensis* Georgi, WRKY, gene family, expression patterns, abiotic stress

## Abstract

The WRKY gene family is an important inducible regulatory factor in plants, which has been extensively studied in many model plants. It has progressively become the focus of investigation for the secondary metabolites of medicinal plants. Currently, there is no systematic analysis of the WRKY gene family in *Scutellaria baicalensis* Georgi. For this study, a systematic and comprehensive bioinformatics analysis of the WRKY gene family was conducted based on the genomic data of *S. baicalensis*. A total of 77 WRKY members were identified and 75 were mapped onto nine chromosomes, respectively. Their encoded WRKY proteins could be classified into three subfamilies: Group I, Group II (II-a, II-b, II-c, II-d, II-e), and Group III, based on the characteristics of the amino acid sequences of the WRKY domain and genetic structure. Syntenic analysis revealed that there were 35 pairs of repetitive fragments. Furthermore, the transcriptome data of roots, stems, leaves, and flowers showed that the spatial expression profiles of WRKYs were different. qRT-PCR analysis revealed that 11 stress-related WRKYs exhibited specific expression patterns under diverse treatments. In addition, sub cellular localization analysis indicated that *SbWRKY26* and *SbWRKY41* were localized in nucleus. This study is the first to report the identification and characterization of the WRKY gene family in *S. baicalensis*, which is valuable for the further exploration of the biological function of SbWRKYs. It also provides valuable bioinformatics data for *S. baicalensis* and provides a reference for assessing the medicinal properties of the genus.

## 1. Introduction

*Scutellaria baicalensis* Georgi is a perennial herb of the Lamiaceae family and a popular Chinese medicine, whose dried root (Huang-Qin) is the primary medicinal component [1]. The main active ingredients of *S. baicalensis* are flavonoids, which possess several vital pharmacological antioxidation, anti-tumor, and anti-virus attributes [2,3,4]. What is even more noteworthy is that it has significant influences on the treatment of COVID-19 [5]. Most transcription factor families can regulate the biosynthesis of secondary metabolites in plants and control the expression levels of some key enzyme genes in the synthesis pathway, which can adjust the content of secondary metabolites. Due to the great medicinal and economic value of *S. baicalensis* [1], the exploration of critical genetic functions has aroused the intense interest of researchers.

Abiotic stresses and hormone treatments can seriously impact various processes during plant growth. For instance, salinity [6], heavy metals [7], low-temperature and drought [8] may greatly reduce crop yields. Plants can almost always respond by mitigating or offsetting the damage caused by diverse stressors. Transcription factors (TFs) are defined as DNA binding proteins that can specifically interact with the *cis*-elements of eukaryotic genes to further activate or inhibit gene transcription. Under adverse conditions, TFs such as MYB [9], bZIP [10] and WRKY [11] play critical regulatory roles in plant growth and development processes, as they typically regulate the expression profiles of stress-responsive genes.

The structure of WRKYs consists of an N-terminal DNA binding domain and a C-terminal zinc finger structure [12]. The amino acid sequence of its DNA binding domain is WRKYGQK; however, occasionally there can be variations such as WRKYGKK and so on [13], and there are two types (C2H2 and C2HC) of zinc finger structures of WRKY [14]. The WRKY gene family comprises three diverse groups according to the number of WRKY domains and zinc finger structures.

The proteins of Group I contain two WRKY domains, whereas both Group II and Group III have single WRKY domains and their zinc finger structures are different. Further, C2H2 belongs to Group II and C2HC belongs to Group III [15]. Due to differences in its primary amino acid sequences, Group II is further divided into five subgroups, designated as II-a, II-b, II-c, II-d, and II-e [16]. WRKYs have been extensively studied in *Arabidopsis thaliana* [17], *Panax ginseng* [18], *Taxus chinensis* [19] and other plants. Over time, they have become the focus of research on the secondary metabolites of medicinal plants.

The WRKY gene family is involved in the regulation of a variety of defense stress responses [20], growth and development [21,22,23], plant hormone signal transduction, and the modulation of pathogen-triggered cellular responses in numerous plant species [24]. For example, the *CaWRKY6* of pepper can bind to and activate the *CaWRKY40* prompter which regulates heat-stress tolerance [25]. The WRKY34 transcription factor negatively mediates the cold sensitivity of mature *Arabidopsis* pollen and might be involved in the CBF signal cascade in mature pollen [26].

*GhWRKY21* plays a negative role in the drought response of cotton; however, the silencing of *GhWRKY21* in cotton dramatically increases drought tolerance [27]. Drought resistance has been observed to be improved in *TaWRKY2*-overexpressing transgenic wheat [28]. The expression of *CgWRKY57* in transgenic plants is higher than that in wild type plants under abscisic acid (ABA) stress [29]. MeJA primes the expression of *PpWRKY70*, and is identified as a transcription activator of PpPAL and Pp4CL via binding to their W-BOX [30]. The above research confirms that WRKYs are of significant importance in the responses of plants to abiotic stresses, which further illustrates their potential functions for enhancing plant stress tolerance.

However, there is currently a lack of systematic analysis of the WRKY gene family in *S. baicalensis*. The importance of *WRKY* genes in the various physiological processes of plants makes it necessary to study their specific roles in *S. baicalensis*. In this work, we identified 77 *SbWRKY* genes from the genome-wide data of *S. baicalensis* based on the highly conserved domains of its family members.

Furthermore, we systematically and comprehensively analyzed the phylogenetic relationships of SbWRKY proteins, chromosome distribution, gene structure, motif composition, gene synteny, and collinearity. In addition, we detected the expression patterns of the stress responsive *SbWRKYs* in different tissues and under abiotic stressors. These results might provide several candidate genes for the further functional investigation of *S. baicalensis*.

## 2. Results

### 2.1. Identification and Chromosome Location of SbWRKY Genes

The hidden Markov model (HMM) of the WRKY domain (PF03106) was employed to search for *S. baicalensis* WRKY genes, and a total of 77 WRKY genes were surveyed (Table S1). Pfam and SMART analyses results revealed that all these proteins contained a complete WRKY domain. These sequences were unevenly distributed on the nine chromosomes of *S. baicalensis*. We designated the genes as *SbWRKY1-SbWRKY75* according to their location on the chromosome, and another two genes (*SbWRKY76* and *SbWRKY77*) did not belong to any chromosome.

**Figure 1:**
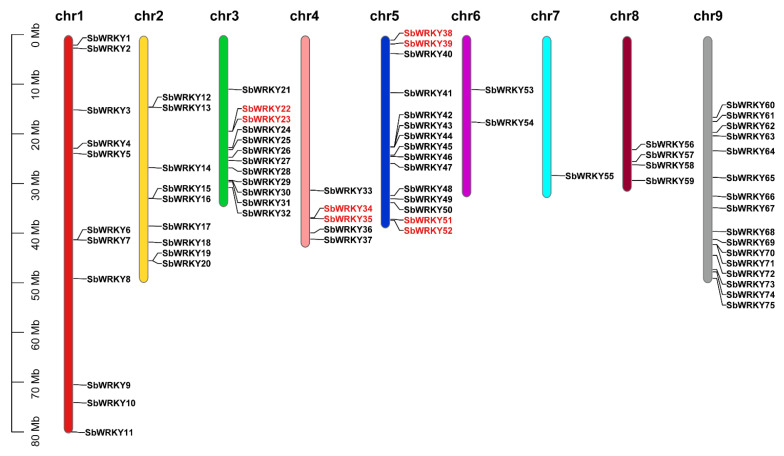
Chromosomal distribution of *SbWRKY* genes. The position of every *SbWRKY* gene can be determined using the left scale. Chr1–Chr9 above the colored bars indicates chromosome (Chr) numbers. The physical location of each *SbWRKYs* is shown, and the gene name is indicated on the right side of each bar as *SbWRKY#* and the red font indicate tandem duplications.

Most of the *SbWRKYs* were abundant on chromosome 9 (16 genes, 20.77%) and chromosome 5 (15 genes, 19.48%) whereas they were negligible on chromosome 7 (1 gene,1.2%). In this case, 12 genes were distributed on chromosome 3, 11 on chromosome 1, and nine on chromosome 2. Five and four *SbWRKYs* were identified on chromosomes 4 and 8, respectively, and two genes were located on chromosome 6 (Figure 1). The diverse sizes and structures of chromosomes may cause these uneven distributions.

We also analyzed the characteristics of the SbWRKYs, including the CDS length, protein molecular weight (MW), isoelectric point (pI), and subcellular location. Among these 77 proteins, SbWRKY22 and SbWRKY23 were identified as the smallest with 58 amino acids, while the largest was SbWRKY71 with 720 amino acids. The MW of the SbWRKYs ranged from 6.844 (SbWRKY22) to 9.81 (SbWRKY71) kDa, and the pI ranged from 4.54 (SbWRKY42) to 9.81 (SbWRKY21). The subcellular localization prediction revealed that 74 SbWRKYs were in the nuclear region, while three proteins (SbWRKY22, SbWRKY23, and SbWRKY42) were located outside the nucleus (Tables S1 and S3).

### 2.2. Multiple Sequence Alignment and Phylogenetic Analysis of SbWRKY Family

To understand the characteristics of the WRKY domains of each SbWRKY protein, Geneious Prime was employed to perform multiple sequence alignment analysis with the amino acid sequences of the WRKY domains in the SbWRKY proteins. The results showed that most of WRKYs contained complete, or close to complete domains in their core sequences. Among them, a few individual amino acids of the conservative motifs of the SbWRKY protein have undergone specific mutations and evolution.

**Figure 2:**
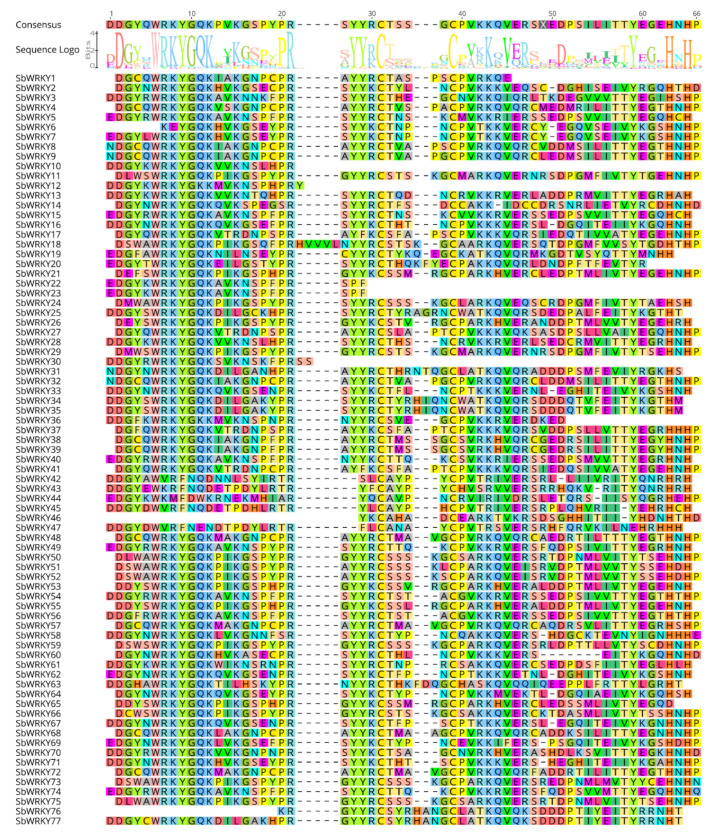
Multiple sequence alignment of the WRKY domain from SbWRKYs. The shading in different shapes and colors indicate the same and conserved amino acid residues, respectively.

For example, the conservative motifs of SbWRKY12 and SbWRKY36 changed from “WRKYGQK” to “WRKYGKK”. In addition, the zinc finger structures of the gene sequences of SbWRKY10, SbWRKY12, SbWRKY30, and SbWRKY36 were missing, and the other SbWRKY sequences all contained the zinc finger structure of C2H2 (Figure 2).

**Figure 3:**
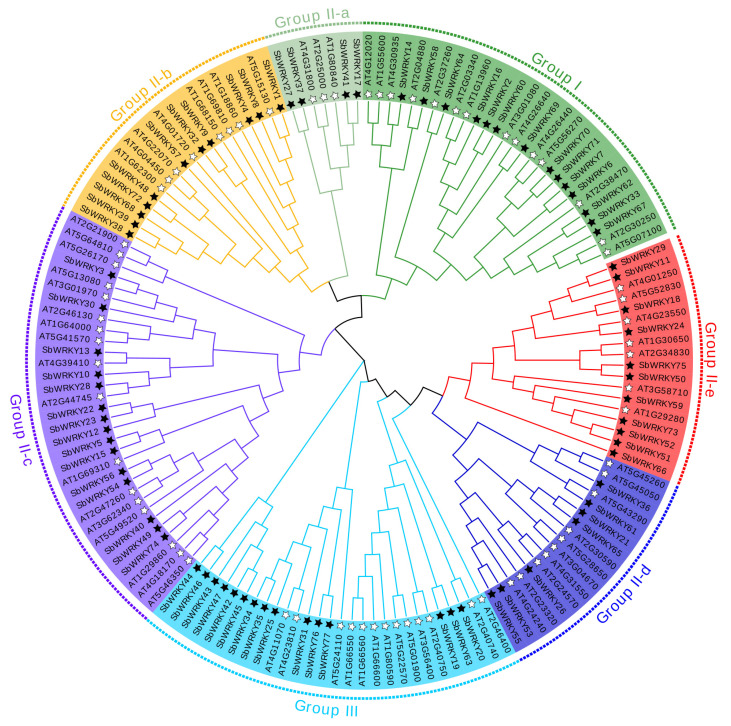
Comparative phylogenetic tree showed the domains relationship of SbWRKYs and AtWRKYs. The unrooted neighbor-joining (NJ) tree was constructed based on the amino acid sequences of WRKYs from *S. baicalensis* (77) and *Arabidopsis* (72) using MEGA-X with 1000 bootstrap replicates. The name of groups (Group I, Group II (Group II-a–Group II-e) and Group III) are shown outside of the circle, indicating different WRKY subgroups. Black star refers to SbWRKYs and white star refers to the AtWRKYs.

To deeply explore the evolutionary relationships of the SbWRKY family members, we selected 72 AtWRKYs of *Arabidopsis* as a reference. MEGA-X was used to cluster and analyze the WRKYs between *S. baicalensis* and *Arabidopsis*. We referred to the classification of AtWRKYs and further distributed the SbWRKYs into three categories: Group I, Group II (II-a, II-b, II-c, II-d, and II-e), and Group III. In addition, Group II was further divided into five subclasses. The classification of SbWRKYs confirmed the diversity of their protein structures, and inferred that various subfamily members might have different regulatory functions. The proteins belonging to Group I contained 15 members with two WRKY domains, Group II contained 48 members, and Group III contained nine members with a single WRKY domain (Figure 3).

These results might be useful for predicting the functions of unknown SbWRKYs based on the functionality confirmed in AtWRKYs or subfamily in *Arabidopsis*. SbWRKY29 in Group II-e might have a similar function as AT4G01250 (AtWRKY22), which might be involved in plant morphogenesis and development that is regulated by auxin and temperature [31]. AT4G39410 (AtWRKY13) can enhance cadmium tolerance by promoting D-CYSTEINE DESULFHYDRASE and hydrogen sulfide production [32], which implies that SbWRKY13 might be related to cadmium tolerance.

### 2.3. Gene Structure and Motif Composition of SbWRKYs

Since the diversity of gene structures can reflect the evolution of gene families, we analyzed the structure of each *SbWRKY* gene to obtain a deeper elucidation of the *S. baicalensis* WRKY family. The data analysis results revealed that *SbWRKY22*, *SbWRKY23*, and *SbWRKY30* contained only one exon. The exon populations in the structures of most *SbWRKY* genes were primarily concentrated at from 2 to 7. The protein members of the same family and their coding genes were highly similar in structure and composition, which verified the reliability of phylogeny.

**Figure 4:**
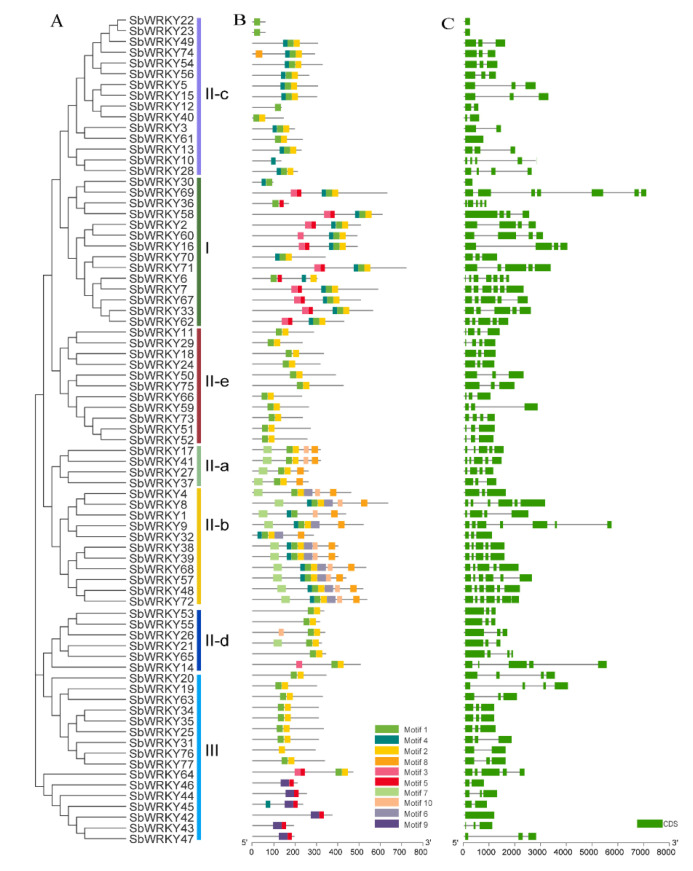
The phylogenetic relationships, gene structure, and composition of conserved motifs in WRKY from *S. baicalensis*. (**A**) The phylogenetic on the left contains 77 WRKY proteins (named SbWRKY1 to SbWRKY77). (**B**) The motif patterns of 77SbWRKY proteins. (**C**) Exon/intron structures of WRKY genes.

To further understand the conservation and diversity of the proteins, the MEME program was used to analyze the conserved motifs of SbWRKYs and predicted the motif composition of the SbWRKY protein. Figure 4 shows that the same group of SbWRKY had highly similar conserved motifs. Motif1 and motif2 were contained in most genes, whereas motif5 was unique to Group I, while motif6, motif7, and motif8 existed only in Group II-b, which meant that they were quite conserved in the SbWRKY protein.

### 2.4. cis-Element Analysis of SbWRKY Genes

**Figure 5:**
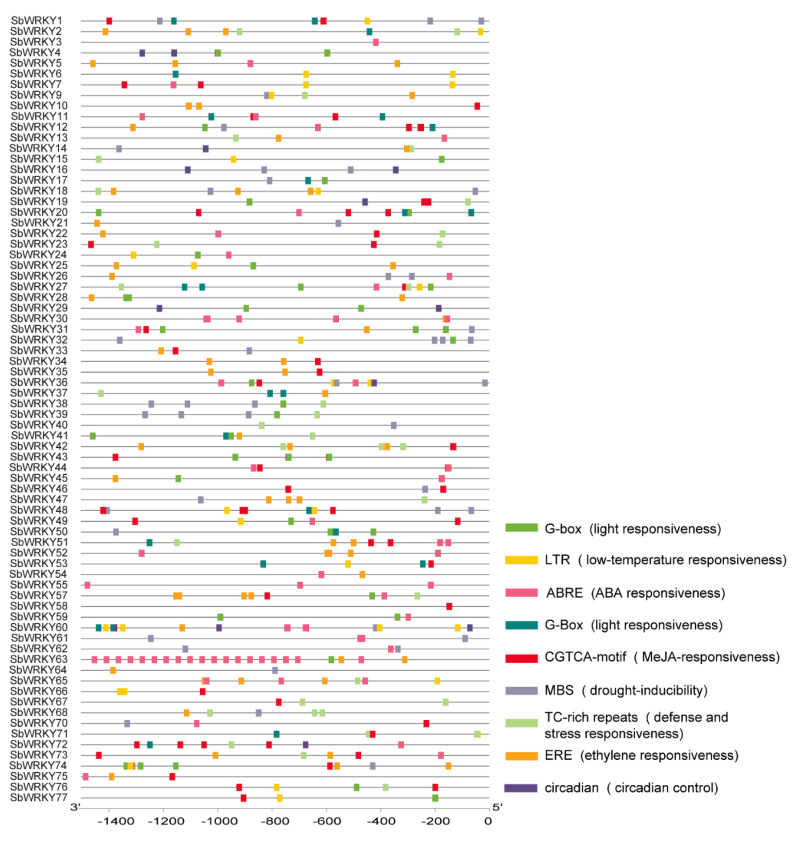
Predicted *cis*-elements that relate to abiotic stress in the SbWRKY prompters. The distribution of *cis*-elements in the 1500 bp upstream promoter regions of *SbWRKY* genes. Different *cis*-elements are represented by different colors.

We analyzed the *cis*-elements of the promoters of the *SbWRKY* genes and focused on the response elements involved in plant growth and development, hormone regulation, and adverse stress. We found that the promoter sequences of 55 *SbWRKY* genes contained the light-responsive element (G-BOX), 52 *SbWRKY* genes contained the ABRE abscisic acid response element, and 39 genes contained the CGTCA-motif methyl jasmonate response element, and 35 family members contained ERE ethylene response elements. There were 27 family members with TC-rich repeats for defense and emergency response and drought-inducible MBS elements, and 22 family members had LTR low-temperature response elements. The *SbWRKY1*, *SbWRKY12*, *SbWRKY27*, *SbWRKY36*, and *SbWRKY74* genes contained six *cis*-elements, and the *SbWRKY60* gene contained seven *cis*-elements (Figure 5 and Table S4). This might mean that these six family members played significant roles in plant growth, development, and stress resistance.

### 2.5. Duplication, Synteny and Ka/Ks Analysis of SbWRKY Genes

**Figure 6:**
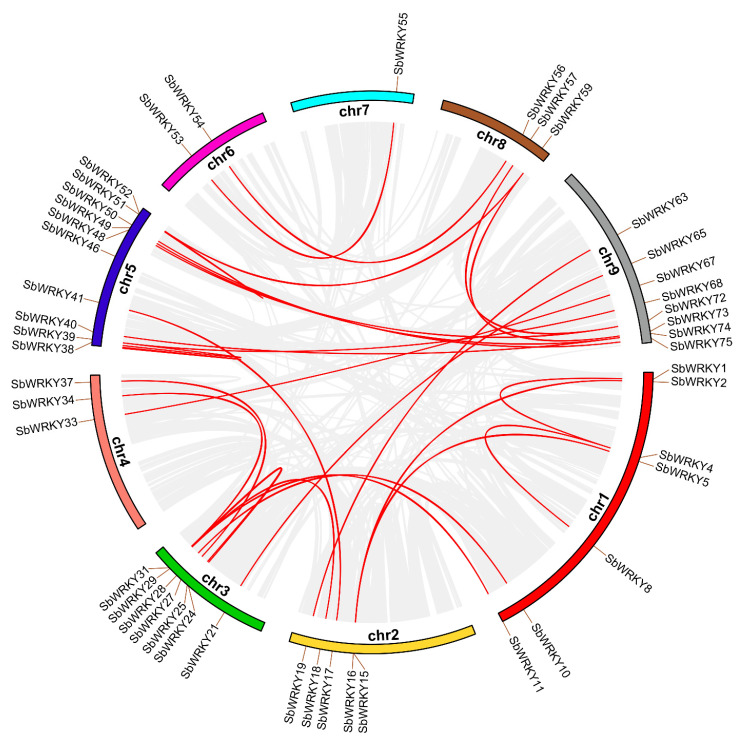
Synteny analysis of interchromosomal relationships of *SbWRKY* genes. All gene pairs and *SbWRKY* gene pairs in the *S. baicalensis* genome were indicated by gray lines and red lines, respectively.

Gene duplication caused by polyploidization or duplication-related tandem and segmental duplication is the main factor in gene family expansion. To clarify the expansion mechanism of the WRKY gene family in *S. baicalensis*, BlastP and MCScanX were employed to identify gene replication modes (tandem and segmented replication). We identified thirty-five pairs of repetitive fragments in the *SbWRKY* genes, and found that some genes that formed tandem repetitive events were from the same subfamily. For instance, *SbWRKY2* and *SbWRKY60* are tandem repeat genes clustered together belonging to Group I; *SbWRKY25* and *SbWRKY31* in Group III are tandem repeat genes (Figure 6).

To explore the origin and evolution of the *SbWRKY* genes, we developed a collinearity map of the *S. baicalensis* WRKY family using three dicotyledonous plants (*Arabidopsis*, potato, and tomato) and three monocotyledonous plants (*Populus trichocarpa*, *Zea mays*, and *Oryza sativa*). There were 65, 55, 54, 46, 17, and 22 *SbWRKY* genes, respectively, that were collinear with the *WRKY* genes of *P. trichocarpa*, potato, tomato, *Arabidopsis*, *Z. mays*, and *O. sativa*.

**Figure 7:**
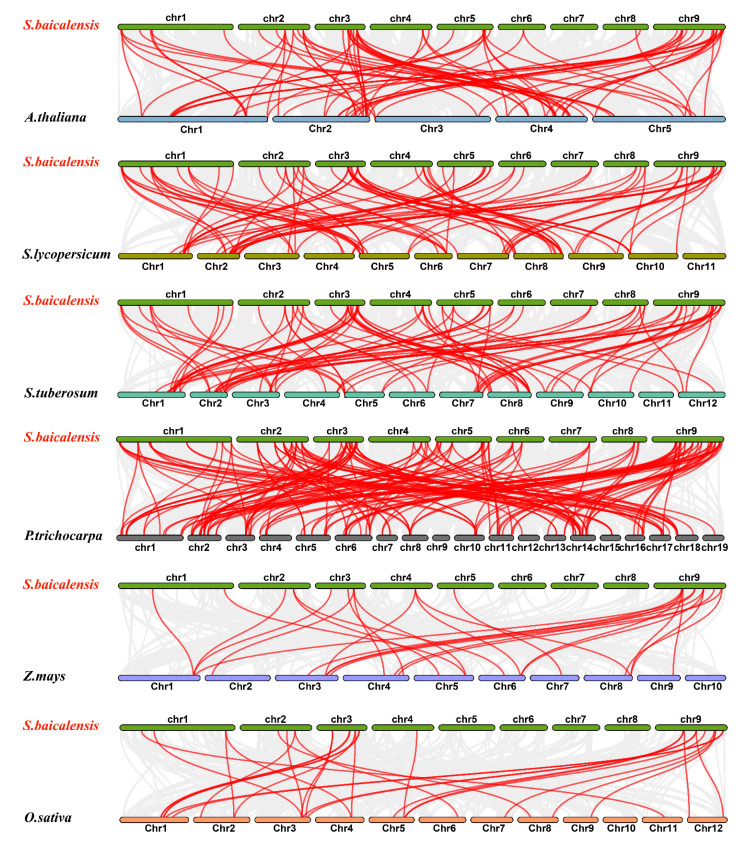
Synteny analysis of SbWRKY genes between and six plant species. The collinear blocks between *S. baicalensis* and other species were showed gray lines. The syntenic WRKY gene pairs between *S. baicalensis* and other species were highlighted in red. The chromosome number was indicated at the top of each chromosome.

A total of 157 collinear WRKY gene pairs of *S. baicalensis* and *P. tomentosa* were identified, followed by *S. baicalensis* and potato (85 pairs), *S. baicalensis* and tomato (83 pairs), *S. baicalensis* and *O. sativa* (29 pairs), *S. baicalensis* and *Z. mays* (27 pairs). We also found that several *SbWRKY* genes (*SbWRKY65* and *SbWRKY17*) had collinear genes with all six selected species, which meant that these genes were likely important in the evolution of the SbWRKY family (Figure 7).

To further investigate whether these homologous *SbWRKYs* underwent selection pressures (purification and positive selection), we calculated the agreed replacement rate (Ks) and non-synonymous replacement rate (Ka) to identify homologous *SbWRKY* gene pairs. Subsequently, the Ka/Ks was calculated to determine whether selection pressure acted on protein-coding *SbWRKY*s. We found that the Ks/Ka ratio of all homologous *SbWRKY* gene pairs was less than 1, indicating that these gene pairs were purified and selected (Table S2).

### 2.6. Analysis of Tissue-Specific Expression Patterns SbWRKY Genes

**Figure 8:**
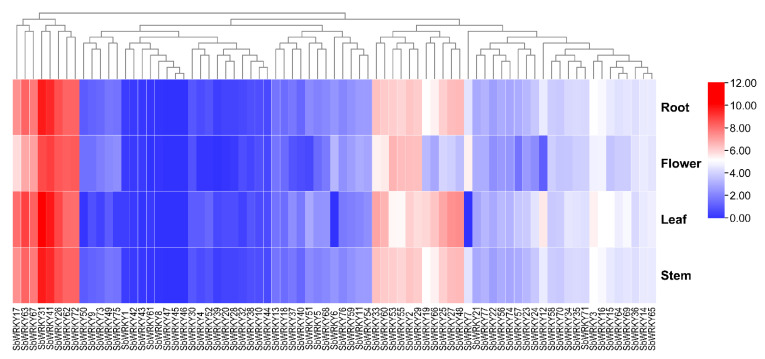
Heat map representation and hierarchical clustering of the *SbWRKY* gene expression profiles in four tissues. Red and blue boxes indicate high and low expression levels of *SbWRKYs*, respectively.

To intensely explore the expression patterns of *SbWRKYs*, we utilized the transcriptome data of four organs (roots, stems, leaves, and flowers) to analyze the transcript abundance (Figure 8 and Table S6). The transcripts of three *SbWRKYs* (*SbWRKY45*, *SbWRKY46*, and *SbWRKY47*) were not detected in the organs, which signified that they might be pseudogenes. In contrast, most *SbWRKY* members were expressed in at least one of the four tested tissues (FPKM > 0). *SbWRKY26* and *SbWRKY41* showed high expression levels in all tissues, particularly in leaves.

### 2.7. Analysis of SbWRKY Gene Expression Patterns of under Different Stress Treatments

**Figure 9:**
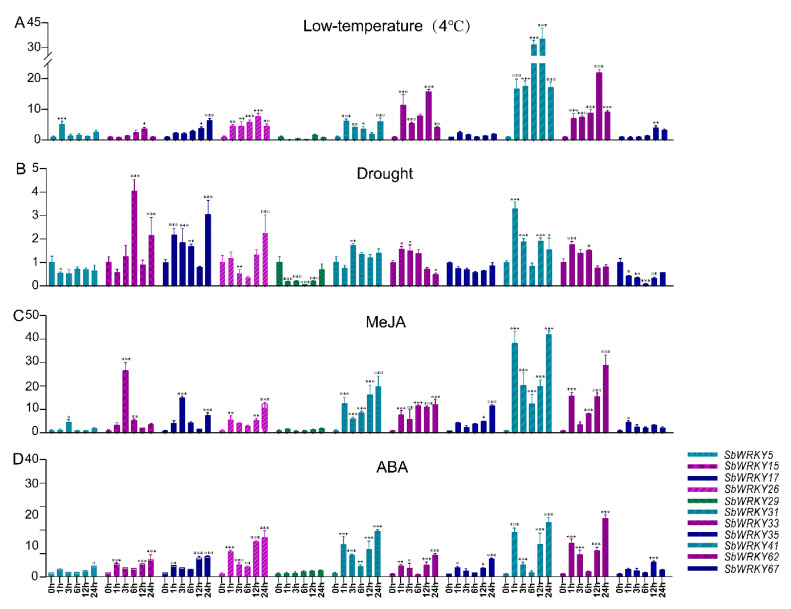
Expression profiles of *SbWRKY* genes under different stress treatments at 0, 1,3,6,12, and 24 h. (**A**) 4 °C. (**B**) Drought. (**C**) MeJA. (**D**) ABA. The data were normalized to the *SbACT2* gene and analyzed with a two-way method using GraphPad Prism, version 8. Asterisks (*p* < 0.05) indicate significant differences compared with the control group (* *p* < 0.05, ** *p* < 0.01, *** *p* < 0.001).

To confirm whether the expressions of *SbWRKY*s were affected by different types of stress treatments, 11 members distributed in different subfamilies were selected based on the analysis results of known stress-related WRKY proteins and *cis*-elements. Subsequently, qRT-PCR was employed further to analyze the effects of different stressors on gene expression. Figure 9 shows the expression levels of selected *SbWRKY*s under four stress conditions (low-temperature, drought, MeJA, and ABA).

Except *SbWRKY29* and *SbWRKY35*, almost all of the genes, comparatively, were significantly up-regulated under low-temperature stress. Among them, *SbWRKY41* was up-regulated ~19-fold at 1 h and 3 h and ~30-fold at 6 h and 12 h after treatment, and the up-regulated increase in gene expression at 24 h was the same at 1 h and 3 h. *SbWRKY62* was upregulated ~22-fold at 12 h. Under drought stress, *SbWRKY5*, *SbWRKY29*, and *SbWRKY67* were down-regulated, while other genes were up-regulated. Interestingly, four genes (*SbWRKY15*, *SbWRKY17*, *SbWRKY28*, and *SbWRKY41*) initially exhibited an up-regulated trend, down-regulation, and then up-regulation with prolonged stress time, where the second up-regulation occurred at 12 h of stress treatment.

Under MeJA stress, except for *SbWRKY29* and *SbWRKY67*, the other genes were up-regulated. *SbWRKY15* was up-regulated ~20-fold at 3 h and *SbWRKY41* was up-regulated ~40-fold at 1 h and 24 h and ~20-fold at 12 h and 12 h following stress treatment. Under ABA stress, except for *SbWRKY29*, the other genes were up-regulated. Among them, *SbWRKY26* was up-regulated ~10-fold at 12 h and 24 h, *SbWRKY31* was up-regulated ~15-fold at 24 h, whereas *SbWRKY41* and *SbWRKY62* were up-regulated ~20-fold at 24 h following the stress treatment.

### 2.8. Subcellular Localization of SbWRKY26 and SbWRKY41

**Figure 10:**
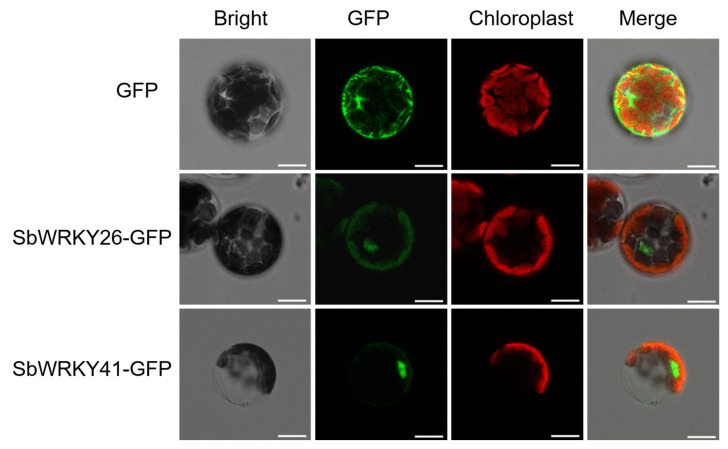
Subcellular localization analysis of SbWRKY26 and SbWRKY41. Subcellular localization of SbWRKY26-GFP and SbWRKY41-GFP in nucleus was confirmed in *Arabidopsis* protoplasts by laser confocal microscopy, with GFP as the positive control (Scale bar: 15 μm).

To reveal the potential functionality of SbWRKY26 and SbWRKY41 in a transcriptional regulation system, HBT-SbWRKY26-GFP-NOS and HBT-SbWRKY41-GFP-NOS fusion protein expression vectors were developed, and an HBT-GFP-NOS vector was used as a positive CK. These vectors were subsequently transferred into *Arabidopsis* protoplasts, where the transient expression was observed under a laser confocal microscope. The GFP positive CK was expressed in the cytoplasm and nucleus, which was consistent with the biological state. The SbWRKY26 and SbWRKY41 experimental group GFPs were mainly expressed in the nucleus (Figure 10), which was consistent with the results of the previous bioinformatic analysis. Similar to many other TFs, SbWRKY26 and SbWRKY41 may play roles in the transcriptional regulation system.

## 3. Discussion

The WRKY gene family is ubiquitous across all plant species and is essential for plant growth and development, as well as the regulation of plant responses to adverse stressors [16,33]. The WRKY gene family has been identified in many species, such as cash crop rice, cucumber [34], *P. trichocarpa* [35], *P. ginseng* [18], and *T. chinensis* [19]. The identification of the WRKY gene family in *S. baicalensis* has not been reported to date, which hinders research into *SbWRKYs* functionality to a certain extent. Therefore, we conducted a systematic bioinformatics analysis of the WRKY gene family of *S. baicalensis*.

For this study, a total of 77 members were identified in the *S. baicalensis* genome. The number of SbWRKYs was less than the identified WRKY genes in *Arabidopsis* (102) and *P. trichocarpa* (104); however, it was more than many species such as *T. chinensis* (61), *P. ginseng* (48), *Isatis indigotica* (64) [36] and *Salvia miltiorrhiza* (61) [37]. This suggests a relatively large number of WRKY family members in *S. baicalensis*, suggesting that repetitive events may occur during genome evolution. It was apparent that two gene replication events (tandem and phased replication) led to gene recombination and amplification that further expanded the WRKY gene family [38]. Relevant studies revealed that gene duplication may be the main driving force behind plant WRKY evolution. The results of multiple sequence alignment and phylogenetic tree analysis indicated that 77 SbWRKY proteins were divided into Group I, Group II (II-a, II-b, II-c, II-d, II-e), and Group III contingent on conserved WRKY domains, which was similar to specific WRKY family proteins in other species.

Previous investigations showed four main WRKY TF lineages in flowering plants, namely Group I+II-c, Group II-a+II-b, Group II-d+II-e, and Group III, which accurately reflected the evolution of the WRKY gene family [39], which was also verified for *S. baicalensis*. Group II-d and Group II-e were found to be divided into two branches, and they belonged to the same large branch in the phylogenetic tree. Although the WRKY domain of the WRKY family was strongly conserved, the SbWRKY protein showed a certain degree of structural difference. As is well known, WRKYGQK is the protein sequence of the conserved domain of the WRKY family; however, several SbWRKY proteins have undergone mutations. For example, the conserved domains of SbWRKY12 (II-c) and SbWRKY36 (II-d) have changed from WRKYGQK to WRKYGKK. Several similar variants have also been found in other species, such as *S. miltiorrhiza* [37], *Broomcorn millet* [40], *Castor Bean* [41]. These mutations may give WRKY various biological functions [42]; however, further experimental verification is required.

Gene duplication primarily includes three forms (tandem, fragment, and genome duplication), which are the main driving force behind the expansion of gene families in plant genomes [43]. There are 35 pairs of fragment duplications in *SbWRKYs*. We also found that some tandem duplication events occurred in the same subfamily of genes, such as *SbWRKY35* and *SbWRKY31* tandem duplications, which both belong to Group III.

We speculated that gene duplication, particularly fragment duplication, is related to the amplification of the SbWRKY family. We also constructed a collinearity map of the SbWRKY family with monocots (*P. trichocarpa*, *Z. mays*, *O. sativa*) and dicots (*Arabidopsis*, tomato, potato). There are 157 collinear gene pairs between *S. baicalensis* and *P. trichocarpa*, 85 pairs between *S. baicalensis* and potato, 83 pairs with tomato, 29 pairs with *O. sativa*, and 27 pairs with *Z. mays*. This indicated that the evolutionary relationship between *S. baicalensis* and *P. trichocarpa* was relatively close.

In addition, the collinear gene pairs between *S. baicalensis* and monocots were fewer than those between *S. baicalensis* and dicotyledons, which may mean that these gene pairs were formed following the differentiation of dicotyledonous and monocotyledonous plants. Furthermore, the analytical results found that several SbWRKY (SbWRKY7, SbWRKY65) proteins and the six selected WRKY protein species possessed collinearity gene pairs, which indicated that they existed before ancestral differentiation.

The exploration of gene expression patterns in different tissues is of great significance for the mining of functional genes. Many studies have found that the WRKY gene is expressed in one or more tissues and plays a vital role in the growth and development of plants. It is well acknowledged that gene expression is intimately related to gene function [44]. This study analyzed the expression patterns of 77 *SbWRKY* genes in the roots, stems, leaves, and flowers of *S. baicalensis*. We found that *SbWRKY* genes in *S. baicalensis* were closely related to their growth and development. Approximately one-third of *SbWRKY* genes were highly expressed in roots, stems, leaves, and flowers (*SbWRKY31*, *SbWRKY41* and other genes).

Similar results have been found in quinoa [45] and cotton [46]. *SbWRKY41* and *SbWRKY31* are highly expressed in roots, and show a close relationship with *AT1G80840* and *AT4G23810*, respectively, in the evolutionary tree. This indicates that *SbWRKY41* and *SbWRKY31* may have similar functions in the regulation of root growth and cell cycles. There are also several other genes that are commonly expressed in roots, stems, leaves, and flowers including *SbWRKY17*, *SbWRKY63*, *SbWRKY67*, *SbWRKY62*, and *SbWRKY72*. This suggests that they might be involved in the regulation of certain biological processes in corresponding tissues; thus, they can be used as candidate genes for research into genetic functionality.

Relevant evidence shows that *WRKY* genes play critical roles in the regulation of plant growth and development, while improving plant tolerance to adversity, including biotic and abiotic stressors [16,33]. We found that almost all *SbWRKY* genes contain stress-related *cis*-elements (e.g., light, heat, cold, drought, and injury), which indicated that these *SbWRKY* genes were involved in various stress responses. Among the 77 *SbWRKY* genes, 52 contained ABRE abscisic acid response elements, and 39 contained CGTCA-motif methyl jasmonate response elements, which implied that *SbWRKY* genes were involved in a variety of plant hormone regulatory pathways.

Further, 27 members contained TC-rich repeats defense and emergency components, and MBS drought inducible components, whereas 22 members contained LTR low-temperature response components. In other species, the *WRKY* gene has been confirmed to be involved in these stress responses. For example, when *Arabidopsis* is under drought stress, *AtWRKY53* accumulates oligosaccharides through sucrose metabolism, which ultimately affects its drought tolerance [47]. A total of 47 *SbWRKY* genes were found to be expressed in the wheat genome under salt stress [48]. Further, *VbWRKY32* positively regulates the transcription level of cold response genes, thereby maintaining membrane stability and improving the survival capacity of *Verbena bonariensis* under cold stress [49].

The latest research reveals that *HbWRKY83* can positively regulate the expression of JA-ethylene and injury-responsive genes in the lactating cells of rubber trees [50]. Several genes in *S. baicalensis* also responded to some abiotic stresses. For instance, *SbWRKY41* gene expression was up-regulated 19 times after 6 h and 12 h of low-temperature induction. Simultaneously, *SbWRKY5*, *SbWRKY29*, and *SbWRKY67* were significantly down-regulated under drought stress, whereas under MeJA stress (for 1 h and 24 h) the expression of *SbWRKY41* was up-regulated 40 times. Under ABA treatment for 24 h, the expressions of SbWRKY41 and *SbWRKY62* genes were observed to increase by 20 times.

## 4. Materials and Methods

### 4.1. Identification, Chromosomal Distribution, Sequence Analysis, Multiple Sequence Alignment and Phylogenetic Analysis of SbWRKYs

We downloaded the hidden Markov model file of the WRKY domain (PF03106) from the Pfam protein family database (http://pfam.xfam.org/family/PF03106, accessed on 6 April 2022) [51], and HMMER3.0 was used to search the *WRKY* genes in the *S. baicalensis* genome assembly of our lab with 0.01 cut-off value default parameters. According to Pfam and SMART analysis, all of these genes contained the complete WRKY domain. The tools provided by the ExPasy website (http://web.expasy.org/protparam/, accessed on 6 April 2022) [52] were used to obtain the sequence length, molecular weight, isoelectric point, and subcellular location prediction of the identified WRKY proteins.

The chromosome positions of all *SbWRKY* genes were determined from the genome annotation file, and TBtools v1.089 (Chengjie Chen et al., China) [53] software was used to draw the chromosome position map.

The domain sequences of the characterized WRKY protein were employed to create multiple sequence alignments with the default parameters of ClustalW [54]. By comparing the predicted coding sequence with its corresponding full-length sequence, the MEME online program (http://meme.nbcr.net/meme/Intro.html/, accessed on 6 April 2022) [55] for protein sequence analysis was used to identify the conservation of the identified SbWRKY protein Motif. The optimized parameters were as follows: the number of repeats was arbitrary, the maximum sequence number was 10, and the optimal width of each motif was between 6 and 100 residues.

For multiple alignment analysis, the SMART program (http://smart.embl.de/, accessed on 6 April 2022) was used to obtain the core sequence of the SbWRKY domain, whereas Geneious v9.1.4 (Biomatters, Auckland, New Zealand) and ClustanXv2.1 (Higgins D.G. et al., Ireland) software further analyzed the core SbWRKY sequence. Simultaneously, we used the WEBLOGO (http://weblogo.berkeley.edu/logo.cgi, accessed on 6 April 2022) online program to show the characteristics of this field. For the phylogenetic tree analysis of the SbWRKYs, we employed the neighbor-joining method to construct a phylogenetic tree with MEGA-X [56] software (1000 bootstraps) and EvolVIEW 2.0 (China national center for bioinformation, Beijing, China) [57] was used for a better image. The *Arabidopsis* WRKY protein sequence was downloaded from the TAIR database (https://www.arabidopsis.org/, accessed on 6 April 2022), and the phylogenetic tree between *S. baicalensis* and *Arabidopsis* was constructed in the same way.

### 4.2. Conserved Motifs, Gene Structure Analysis, cis-Elements, Ka/Ks and Synteny Analysis of SbWRKY Proteins

TBtools [53] software was used to display motif results of the XML file obtained from MEME. We also used TBtools software combined with the genomic sequence to show the genetic structures of *SbWRKYs*.

BioEdit (Borland, Scotts Valley, CA, USA) software was used to obtain the 1500 bp promoter sequence located upstream of the gene in the whole genome data of *S. baicalensis*, and search for potential *cis*-elements of *SbWRKYs* in the PlantCare database [58]. We used TBtools to visualize the *cis*-elements of the promoter. To verify whether there was positive selection in the evolution of *SbWRKY* genes, the online websites of Clustal Omega (https://www.ebi.ac.uk/Tools/msa/clustalo/, accessed on 6 April 2022) and PAL2NAL (http://www.bork.embl.de/pal2nal/, accessed on 6 April 2022) were used to calculate the synonymous substitution rate (Ks) and non-synonymous substitution rate (Ka) values of homologous gene pairs and their amino acid sequences in the SbWRKYs.

The Multiple Collinearity Scan toolkit (MCScanX) [59] was utilized to check gene duplication events with default parameters. To explore the syntenic relationships between *S. baicalensis* and other species, the Dual Systeny Plot of TBtools was used to map synteny between *S. baicalensis* and the other selected species (*P. trichocarpa*, potato, tomato, *Arabidopsis*, *Z. mays*, and *O. sativa*) and Advanced Circos of TBtools was used to show the intraspecies gene duplication of *S. baicalensis*.

### 4.3. Subcellular Localization Analysis of SbWRKY26 and SbWRKY41

*SbWRKY26* and *SbWRKY41* were cloned from cDNA obtained by reverse transcription of *S. baicalensis*, which were obtained as previously described via SbWRKY26-F/R and SbWRKY41-F/R primers. The amplified products were then inserted into a TOPO vector (Vazyme) and verified by DNA sequencing. The amplification of SbWRKY26 and SbWRKY41 was performed using GFP-SbWRKY26-F/R and GFP-SbWRKY41-F/R primers (Table S5). The amplified PCR products of the SbWRKY41 and HBT-GFP-NOS vectors were digested with *BamH I* and *Sma I*, whereas the products of the SbWRKY26 and HBT-GFP-NOS vectors were digested using *Sac II* and *Kpn I*. Subsequently, the SbWRKY26, SbWRKY41, and HBT-GFP-NOS vector products were ligated with DNA ligase to create HBT-SbWRKY26-GFP-NOS and HBT-SbWRKY41-GFP-NOS fusion protein expression vectors.

Mesophyll protoplasts were then isolated from *Arabidopsis* and transformed as previously described [60,61]. The transformed protoplasts were grown at 21 °C for 12 to 16 h, after which high resolution confocal laser microscopy (Leica TCS SP5, LEICA, Wetzlar, Germany) was used to observe the subcellular localization of fusion proteins and to take images of GFP, chlorophyll, and bright field channels (excitation at 488 nm; emission at 500–535 nm), respectively, which were then merged.

### 4.4. Plant Material and Treatments

*S. baicalensis* seeds collected from Yangcheng County, in Shanxi Province were soaked in water for 12 h to soften the seed coat, and then placed at room temperature 23 ± 2 °C under natural light (16 h light/8 h dark) and 60–80% humidity conditions to facilitate germination. Subsequently, three-month-old seedlings were subjected to different abiotic stresses. For the low-temperature treatment the three-month-old seedlings in the pots were exposed to 4 °C, whereas for the drought treatment the seedlings were subjected to different treatments for 0 h (as the control sample),1 h, 3 h, 6 h, 12 h, and 24 h by pouring 20% PEG6000 on the roots of the seedlings growing in the soil mixture containing pots.

For the hormonal treatments, the surface of the aboveground part of seedlings was sprayed with 100 μM MeJA and ABA, respectively. Plants under the different stress conditions were collected at the corresponding five time periods (0 h, 1 h, 3 h, 6 h, 12 h, and 24 h), immediately frozen in liquid nitrogen, and stored at −80°C for subsequent RNA extraction. To study the expression patterns of *SbWRKYs* genes under different stressors, we selected 11 *SbWRKY* genes for further qRT-PCR analysis.

### 4.5. Extraction of Total RNA and Synthesis of cDNA

RNA was extracted from the whole Plant of *S. baicalensis* using Plant Rapid RNA Extraction Kit RP3501 (BioTeke, Wuxi, China), while the RNA concentration and ratio (A260/A280 and A260/A230) were measured via a NanoDrop2000c spectrophotometer (Thermo Science, Waltham, MA, USA), after which the RNA integrity was observed with 1% agarose gel. For cDNA synthesis, a total of 1 μg RNA was used for reverse transcription in a volume of 20 μL following the instructions of the PrimeScriptRT kit (Highland City, Kusatsu City, China).

### 4.6. RNA-Seq Expression and qPCR Analysis

Four different tissues (roots, stems, leaves, and flowers) of *S. baicalensis* were collected during the flowering period for transcriptome sequencing, and the tissue-specific expression patterns of *SbWRKYs* were further detected. The method used to calculate the transcript abundance of *SbWRKYs* was through its estimation according to the number of fragments per base in the exon model (FPKM) per million mapped reads. Real-time fluorescent quantitative PCR (qPCR) used a Roche LightCycler 96 system (Roche Diagnostics GmbH, Basel, Switzerland) with ChamQTM SYBR^®^ qPCR Master Mix (Roche), where the PCR reaction conditions were 95 °C for 30 s, 95 °C for 5 s, and 60 °C for 30 s, for a total of 45 cycles. Each reaction had three biological and technical replicates, using 20-fold diluted cDNA as a template. The 2^−∆∆CT^ method was employed to calculate the corresponding expression of *SbWRKYs*.

## 5. Conclusions

This study lays the foundation for the functional analysis of the role of the *SbWRKY* gene in *S. baicalensis*. We identified 77 *SbWRKY* genes and conducted a comprehensive analysis in terms of phylogeny, genetic structure, conserved domains, homology, collinearity, and gene expression patterns. In addition, we predicted the potential functions of several SbWRKY proteins through phylogenetic comparison and gene expression profiling. This study reveals a basic understanding of the characteristics of the SbWRKY gene family and provides valuable information for enhancing the growth regulation and defense capabilities of *S. baicalensis*.

## Supplementary Materials

The following supporting information can be downloaded at: https://www.mdpi.com/1422-0067/23/8/4225/s1.
